# *Annona muricate* Extract Supplementation Contributes to Improve Aberrant Multi-Organ Energy Metabolism via Muscle–Brain Connectivity in Diabetic Mice

**DOI:** 10.3390/nu15112559

**Published:** 2023-05-30

**Authors:** Heaji Lee, Sun Yeou Kim, Yunsook Lim

**Affiliations:** 1 Department of Food and Nutrition, Kyung Hee University, 26 Kyunghee-Daero, Dongdaemun-Gu, Seoul 02447, Republic of Korea; ji3743@khu.ac.kr; 2 Gachon Institute of Pharmaceutical Science, Gachon University, 191 Hambakmoero, Yeonsu-gu, Incheon 21936, Republic of Korea; sunnykim@gachon.ac.kr

**Keywords:** type 2 diabetes mellitus, myokines, hepatokines, energy metabolism, skeletal muscle, brain, sarcopenia, cognitive dysfunction

## Abstract

Type 2 diabetes mellitus (T2DM) is related with the incidence of sarcopenia and cognitive impairment that reduces quality of life in the elderly. Recent evidence has demonstrated that sarcopenia is associated with cognitive dysfunction, and muscle-derived endocrine factors might contribute to cognitive function by the skeletal muscle–brain endocrine loop. This study investigated the beneficial effects of *Annona muricata* (AM, graviola) on multi-organ energy metabolism with muscle–brain connectivity via brain function-related myokines in mice. Body composition, fasting blood glucose level, insulin, HbA1c%, histopathological changes, and the protein levels of insulin-signaling, energy metabolism, neuroprotection, inflammation, and protein-degradation pathways were measured. AM extract (AME) treatment selectively enhanced insulin signaling in the skeletal muscle and hippocampus of T2DM mice. Furthermore, AME treatment effectively increased muscle-derived fibroblast growth factor 21 (FGF21), cathepsin-B (CTSB), irisin, brain-derived neurotrophic factor (BDNF), and liver-derived FGF21 that contribute to whole-body energy homeostasis. In particular, AME increased the levels of circulating myokines (FGF21, BDNF, irisin, and CTSB), and these were accordance with the hippocampal neurotrophic factors (BDNF and CTSB) in T2DM mice. In conclusion, we suggest that AME would be a potential nutraceutical for improving the energy metabolism associated with muscle–brain connectivity via brain function-related myokines in T2DM.

## 1. Introduction

Type 2 diabetes mellitus (T2DM) patients are known to have multi-organ dysfunction accompanied by nephropathy, retinopathy, sarcopenia, neuropathy, and non-alcoholic fatty liver disease [1,2]. Among them, sarcopenia and cognitive dysfunction are two of the most common conditions in the elderly [3,4]. In T2DM, skeletal muscle damage is accelerated due to glucose toxicity, insulin resistance (IR), and oxidative stress [1]. Furthermore, a lot of evidence has reported the association pathway between T2DM and neurological deficits [4]. Metabolic disorders decreased cerebral blood flow and partially mediated the lowering of immediate memory function [4]. In diabetic patients, there were abnormalities in neuroimaging such as alternations in the microstructure and neuronal circuitry in the striatum [5]. These neurobiological changes seen in the metabolic disease are associated with oxidative damage and neuro-inflammation as well as increased misfolded proteins and cellular senescence [5]. These factors could be major underlying mechanisms for chronic metabolic diseases causing physical disability [4]. Given that T2DM patients have increased susceptibility for sarcopenia and cognitive impairment, understanding the association between them is important in T2DM [1,5]. Although the molecular mechanisms between sarcopenia and cognitive dysfunction remain unclear, common risk factors might cause the incidence of multi-organ damage in T2DM [2].

In epidemiological studies, it is well-documented that sarcopenia has been linked to cognitive decline in diabetes [6,7,8]. In patients with T2DM, reduced skeletal muscle mass has been related with cognitive impairment [6]. There was an association between sarcopenia and mild cognitive impairment elderly patients with diabetes (odds ratio = 2.96, *p* = 0.032) [7]. Recent studies have demonstrated that exercise-induced myokines contribute to a direct interaction between muscles and the brain [9,10,11]. In our previous study, exercise enhanced the production of brain function-related myokines including BDNF in OB mice [10]. Furthermore, we examined that liver and skeletal muscle-derived molecules were associated with the expression of neurotropic factors in the brains of T2DM mice [11].

From this evidence, it is postulated that liver/muscle-derived hepatokines/myokines might contribute to cognitive function. Hepatokines/myokines are liver/muscle-derived cytokines and chemokines which regulate whole-body metabolism by autocrine manner in muscle/liver tissue as well as paracrine or endocrine action with other tissues [9]. These biomarkers work together in the body to form complex networks of action in various tissues that can impact a variety of physiological disorders. Therefore, abnormality of metabolic homeostasis in endocrine organs influences the production and release of myokines/hepatokines, thereby compromising their proper signaling to multiple organs, including the brain.

In T2DM, metabolic disorders progressively disrupt the cellular and molecular mechanisms of the muscle–liver axis, resulting in energy deficits. In particular, IR leads to aberrant energy metabolism via the fibroblast growth factor 21 (FGF21)-dependent sirtuin (SIRT)1/peroxisome proliferator-activated receptor γ coactivator 1α (PGC1α) pathway [12,13,14,15,16]. FGF21 regulates a lot of metabolic pathways such as glucose and lipid metabolism via insulin secretion [14]. FGF21 utilizes the conventional FGF receptor-1 (FGFR1) and the co-receptor β-klotho to control endocrine and paracrine communication networks that physiologically regulate the metabolic pathway. FGF21 increases the NAD+ levels that subsequently activate SIRT1 and deacetylate its downstream target, PGC-1α. The activation of SIRT1/PGC1α by FGF21 increases brain function-related hepatokines (FGF21 and irisin) and myokines (FGF21, irisin, BDNF, and CTSB. Recent studies have suggested that these muscle-derived mediators pass through the brain blood barrier (BBB), directly enhancing neuroprotective markers including irisin and BDNF [11]. They mediate neurotropic signaling to modulate neuronal homeostasis while favoring neurogenesis and synaptic plasticity, suggesting a possible mechanism for enhancing cognitive function [9].

In this regard, peripheral energy metabolism might contribute to cognitive function directly/indirectly by endocrine factors released from peripheral tissues. By regulating this pathway, the metabolic capacity of skeletal muscle and the liver can robustly improve the metabolic function of multiple organs, including the brain. In addition, these biomarkers have been largely associated with early metabolic changes via multi-organ crosstalk and serve as potential therapeutic targets that may help combat T2DM-associated metabolic disorders. Moreover, as T2DM patients are susceptible to complications in various organs simultaneously, intervention targeting multi-organs is needed in order to prevent/alleviate multi-organ complications induced by T2DM.

In this respect, *Annona muricata* (AM, graviola) is expected to be nutraceutical to attenuate diabetic complications. AM is a functional food and is traditionally used as a tea for therapeutic purposes. AM is rich in natural phytochemicals such as alkaloids, phenols, acetogenins, flavonoids, and vitamins [17,18]. These compounds demonstrate several biological activities including antioxidant, antihypertensive, antibacterial, antidiabetic, and anticancer effects [19]. AM is especially known for its hypoglycemic effect and anti-inflammatory activity in diabetic patients [17]. In our previous study, AME enhanced hepatic energy metabolism and autophagy, which improve hepatic function in T2DM mice [20]. Furthermore, graviola leaves attenuated behavior changes in diabetes, although the protective mechanism on the brain is not clear [21].

However, the effects of dietary intervention, including nutraceuticals as well as AM, on multi-organ abnormality accompanied by sarcopenia and cognitive impairment in T2DM have not been extensively investigated. In addition, no research has shown a crosstalk or endocrine factors focusing on the therapeutic effects of nutritional intervention. An integrated approach related to myokines/hepatokines is needed to develop a new intervention for various metabolic diseases. The objective of this study is to examine the beneficial effects of the ethanol extract of AM (AME) on multi-organ energy metabolism with muscle–brain connectivity in T2DM. Particularly, we tried to investigate the mechanisms of myokines/hepatokines derived from muscle/liver, which are associated with neuroprotection by AME supplementation.

## 2. Materials and Methods

### 2.1. Annona Muricata (AM) Extract

AM leaves were purchased in dried form (DS International, Jeungpyeong-gun, Chungcheongbuk-do, Republic of Korea). Dried AM leaves were extracted twice in 50% ethanol at 25 °C. The extract was prepared into powder form through solvent evaporation, Then, the samples were evaporated followed by freeze-drying. Finally, the powder form of the extract was kept at 4 °C until further use.

### 2.2. Animals and Experimental Design

Four-week-old male C57BL/6 mice (Raon Bio, Yongin-si, Gyeonggi-do, Republic of Korea) were housed with water ad libitum. They were kept on a 12 h light and dark cycle and maintained at 22 °C. Induction of T2DM was performed according to previous studies [11,20]. All mice were first randomly divided into two groups: the control group that was fed a 10% kcal control diet (D12450J; Research Diets, New Brunswick, NJ, USA) and the type 2 diabetes mellitus group, which was fed a 60% kcal high-fat diet (HFD) (D12492; Research Diets, New Brunswick, NJ, USA). After continuous HFD feeding for 4 weeks, mice were injected with 60 mg/kg streptozotocin solution (STZ, dissolved in a citric acid buffer, 0.01 M, pH 4.5) once a week for 2 consecutive weeks [22].

**Figure 1:**
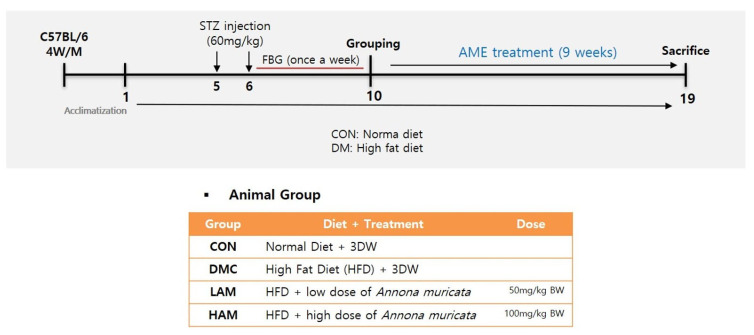
Experimental design.

After the second injection, fasting blood glucose (FBG) was detected using a glucose meter (LifeScam Omc., Milpitas, CA, USA) every week. Mice with FBG > 250 mg/dL at least twice in four weeks were judged as diabetic model mice. After 9 weeks of diabetes induction, the animals were divided into 4 experimental groups as follows: CON, normal control group; DMC, diabetes mellitus control group; LAM, diabetic mice supplemented with a low dosage of AME (50 mg/kg BW); HAM, diabetic mice supplemented with a high dosage of AME (100 mg/kg BW) (n = 11~12 per group) [20]. Ethanol extract of AM was suspended in distilled water and orally gavaged every day for 9 weeks. The concentration of each stock solution was 12.5 mg/mL (low dosage of AME, LAM) and 25 mg/mL (high dosage of AME, HAM), respectively. Body weight, food intake, and FBG level were measured once a week (Figure 1). All experimental protocols were approved by Kyung Hee University for animal welfare (KHSASP-20-060, approval date: 13 March 2020).

### 2.3. Measurement of Body Composition

Dual-energy X-ray absorptiometry (DXA; InAlyzer, Medikors, Seungnam, Republic of Korea) was used to analyze the composition of the body after the treatment was completed. Mice were anesthetized with ketamine and placed in a cabinet X-ray system [23].

### 2.4. Hemoglobin A1c (% HbA1c) and Insulin Level

HbA1c% and plasma insulin were measured using commercial kits according to the manufacturer’s instruction (Crystal Chem., Downers Grove, Elk Grove Village, IL, USA; RayBiotech, Inc., Norcross, GA, USA).

### 2.5. Homeostasis Model Assessment of IR (HOMA-IR) Level

The HOMA-IR value was calculated as follows: HOMA-IR = fasting glucose (mmol/mL) × fasting plasma insulin (µU/mL)/22.5.

### 2.6. Histological Analysis

The tissue samples (skeletal muscle (gastrocnemius), the liver, and the whole brain) were paraffin-embedded and sectioned at 4 µm-thickness. The sections were stained with hematoxylin and eosin to evaluate the histological changes in skeletal muscle and brain tissue [24].

### 2.7. Protein Extraction and Western Blot Analysis

Western blot analysis was performed with the same method used in a previous study [25]. Cytosolic and nuclear proteins were extracted from the liver, gastrocnemius tissue, and hippocampus with lysis buffers containing protease/phosphatase inhibitor, cytosol-extracting buffer. A total of 30 μg of protein for each group was used for western blot analysis. The extract was separated by 8~12% SDS-PAGE gel electrophoresis then transferred to a polyvinylidene fluoride membrane (Millipore, Billerica, MA, USA). Then, it was incubated overnight with primary antibodies and probed with the respective secondary antibody reagent (Biorad, Hercules, CA, USA). The bands were adjusted by the band levels of α-tubulin (cytosol) or PCNA (nucleus) [25].

Antibody list: pAkt (sc-81434), Akt (sc-514032), GLUT4 (sc-53566), GLUT2 (sc-518022), FGF21 (sc-81946), KYN (sc-69890), PPARα (sc-398394), FGFR1 (sc-57132), CTSB (sc-365558), PGC1α (sc-517380), Foxo3a (sc-48348), Atroin-1 (sc166806), Murf-1 (sc-398608) (Santa Cruz Biotechnology, CA, USA, 1:200), IRS-1 (#2382), pIRS-1 (#2381), mTOR (#2972), pmTOR (#2971), LC3 (#2775), (#2535), AMPK (#2532) (cell signaling technology, MA, USA, 1:2000), FNDC5 (ab131390), BDNF (ab108319), β-klotho (ab127879) (Abcam, Cambridge, MA, USA, 1:10,000), α-tubulin (T5168, Signa Aldrich, MO, USA, 1:4000), PCNA (610665, Enzolife science, Farmingdale, NY, USA, 1:1000).

### 2.8. Statistical Analysis

All data are presented as means ± SEM. The significance of differences was analyzed by one-way ANOVA. A probability level of *p* < 0.05 was considered statically significant. All statistical analyses were performed using SPSS (version 23.0 for Windows, IBM, New York, NY, USA).

## 3. Results

### 3.1. Effects of AME Treatment on Body Composition, Food Intake, and Glycemic Regulation in T2DM Mice

After 9 weeks supplementation of AME, the FBG and insulin levels of the LAM group were lower than those of the DMC group. Furthermore, AME supplementation decreased HbA1c% at both doses, and insulin levels at the low dose compared to those of the DMC group. In addition, HOMA-IR was decreased in the LAM group, whereas it was increased in the DMC group. AME did not normalize body composition and food intake in the diabetic mice (Table 1).

### 3.2. Effects of AME Treatment on Skeletal Muscle and Brain Morphology in T2DM Mice

**Figure 2:**
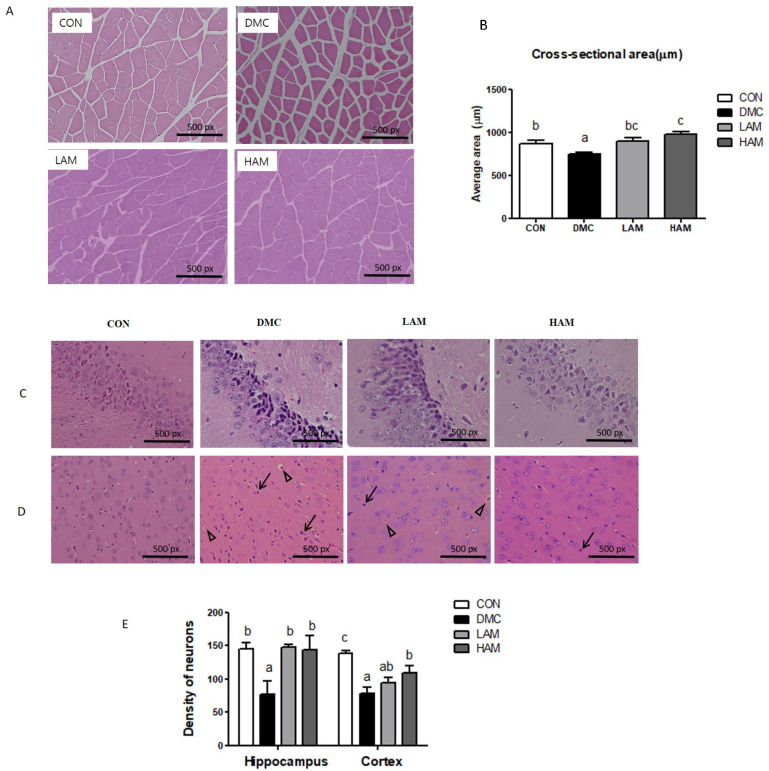
Effects of AME treatment on skeletal muscle and brain morphology (×200) in T2DM mice (↑: karyopyknosis, ∆: acidophilic necrosis). Skeletal muscle (×200) (**A**), Cross-sectional area (**B**), hippocampus (×200) (**C**), cortex (×200) (**D**), and density of neurons (**E**). Values are means ± SEM. There is no significant difference between values with the same superscript letters within groups (n = 9 per group).

AME treatment increased the average cross-sectional area, which was decreased in the DM mice (Figure 2A,B).

AME treatment ameliorated the neuronal damage including nuclei pyknosis in T2DM mice. Furthermore, both doses of AME treatment normalized the number of surviving neurons in the hippocampus of T2DM mice. In addition, the high dose of AME treatment enhanced the number of surviving neurons in the cortex of T2DM mice (Figure 2C–E).

### 3.3. Effects of AME Treatment on Insulin-Signaling-Related Markers in T2DM Mice

**Figure 3:**
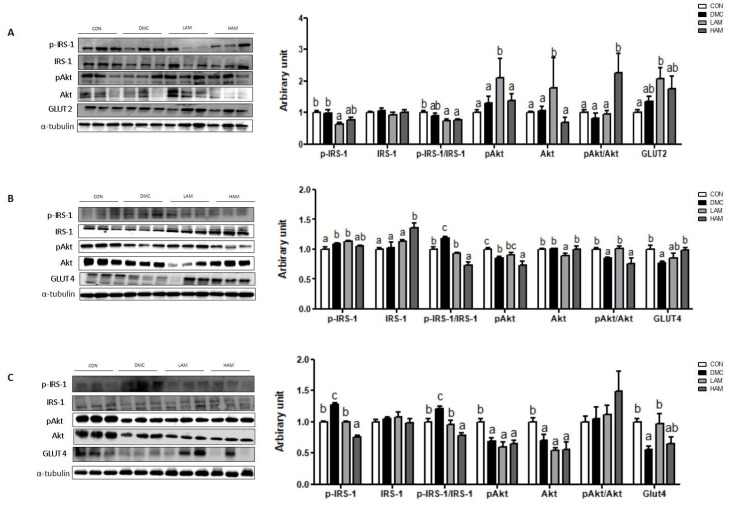
Effects of AME treatment on insulin-signaling-related markers in the liver (**A**), skeletal muscle (**B**), and hippocampus (**C**) in T2DM mice. Values are means ± SEM. There is no significant difference between values with the same superscript letters within groups (n = 9 per group).

In hepatic tissue, the protein level of p-IRS-1 (Ser) was decreased in the LAM group compared to the DMC group. The ratio of pAkt/Akt was increased in the HAM group compared to the DMC group. In addition, the protein level of GLUT2 was not changed by AME supplementation in T2DM mice (Figure 3A).

In skeletal muscle, both doses of AME treatment decreased the ratio of p-IRS-1 (Ser)/IRS-1 in T2DM mice. Furthermore, low dose of AME treatment increased the ratio of pAkt/Akt and high dose of AME treatment increased the protein level of GLUT4 in T2DM mice (Figure 3B).

In hippocampus, both dose of AME treatment decreased the protein level of p-IRS-1 (Ser) in T2DM mice. Furthermore, low dose of AME treatment significantly increased the protein level of GLUT4 in T2DM mice. The protein levels of pAkt and Akt were not normalized by AME treatment in T2DM mice (Figure 3C).

### 3.4. Effects of AME Treatment on Energy Metabolism-Related Markers in T2DM Mice

Energy metabolism in the liver, skeletal muscle, and hippocampus was impaired in T2DM mice. AME supplementation selectively enhanced energy metabolism pathway.

**Figure 4:**
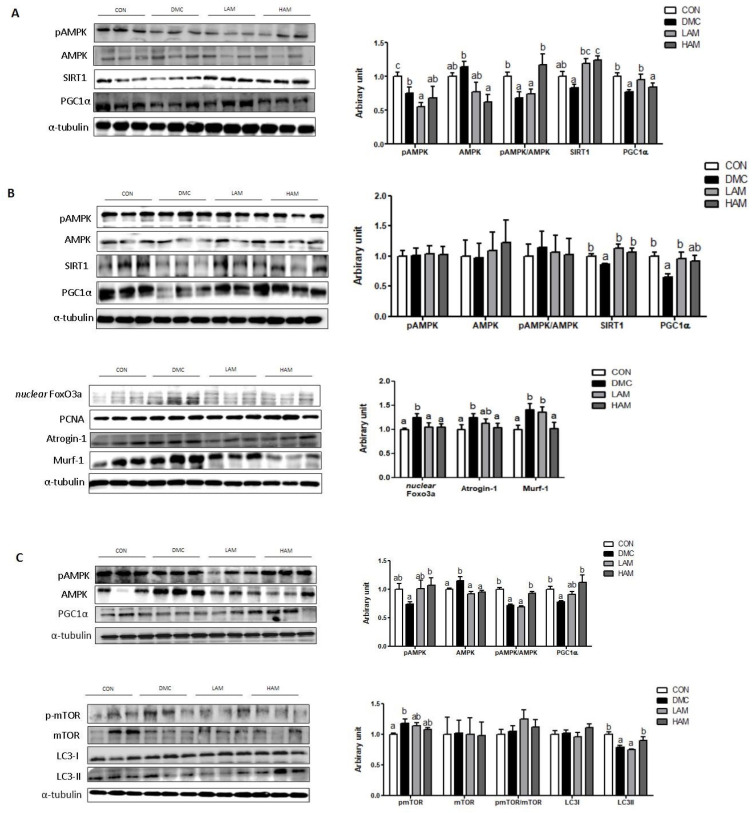
Effects of AME treatment on energy metabolism-related markers in the liver (**A**), skeletal muscle (**B**), and hippocampus (**C**) in T2DM mice. Values are means ± SEM. There is no significant difference between values with the same superscript letters within groups (n = 9 per group).

In the hepatic tissue of T2DM mice, AME supplementation increased the ratio of pAMPK/AMPK and PGC1α at a high dose and low dose, respectively. In addition, both doses of AME supplementation increased the protein level of SIRT1 (Figure 4A).

In the skeletal muscle of T2DM mice, AME treatment increased the protein level of PGC1α at the low dose and SIRT1 regardless of the dose. Furthermore, both doses of AME treatment decreased the protein level of nuclear Foxo3a. In addition, the high dose of AME treatment decreased the protein levels of Atrogin-1 and Murf-1 (Figure 4B).

In the hippocampus of T2DM mice, the high dose of AME treatment increased the protein level of pAMPK and the pAMPK/AMPK ratio. In addition, the high dose of AME treatment increased the protein level of PGC1α. AME treatment did not normalize the protein level of pmTOR. The protein levels of mTOR and LC3I were not different among the groups. The protein level of LC3II was increased by the high dose of AME treatment (Figure 4C).

### 3.5. Effects of AME Treatment on Hepatokines and Myokines Associated with Neuroprotection in T2DM Mice

**Figure 5:**
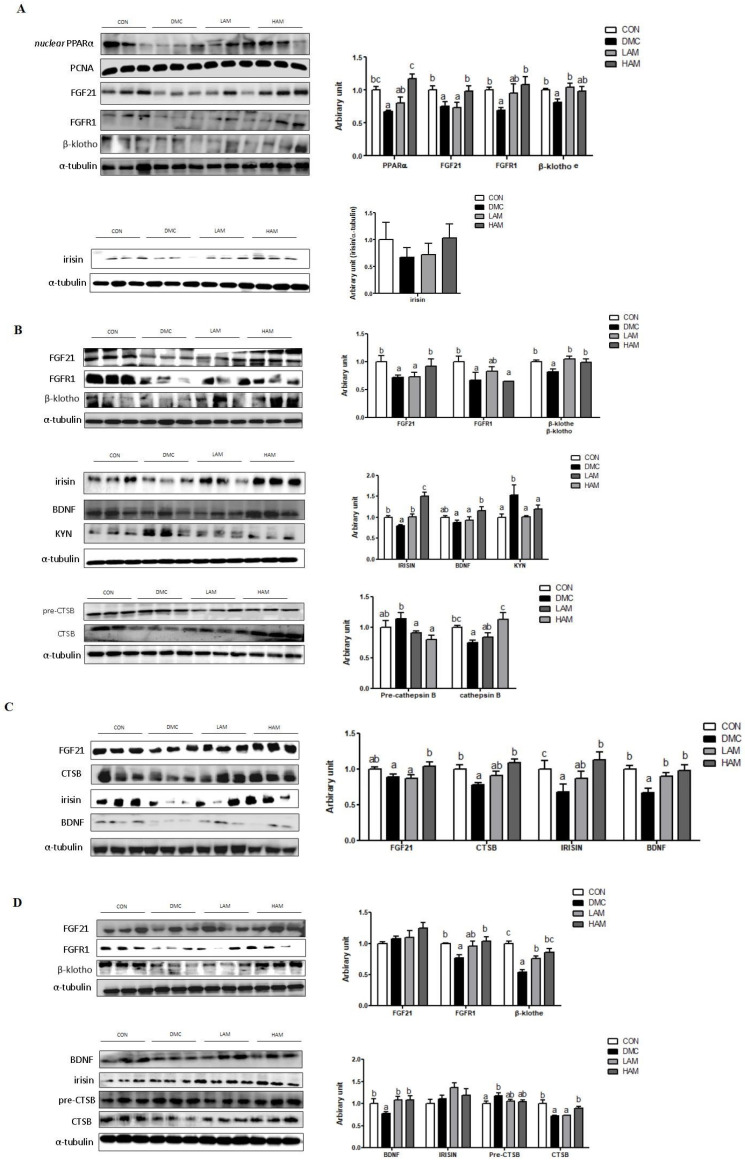
Effects of AME treatment on hepatokines and myokines associated with neuroprotection in the liver (**A**), skeletal muscle (**B**), plasma (**C**), and hippocampus (**D**) in T2DM mice. Values are means ± SEM. There is no significant difference between values with the same superscript letters within groups (n = 9 per group).

In the hepatic tissue of T2DM mice, the high dose of AME increased the protein levels of hepatic FGF21 and FGFR1 in T2DM mice. Furthermore, the low dose of AME treatment increased the protein level of β-klotho. The protein level of irisin was not different among the groups (Figure 5A).

In the skeletal muscle of T2DM mice, the high dose of AME treatment increased the protein level of skeletal muscle FGF21. Furthermore, both doses of AME treatment increased the protein levels of β-klotho, irisin, and kynurenine (KYN). The high dose of AME treatment increased the protein levels of BDNF and CTSB (Figure 5B).

In the plasma of T2DM mice, the high dose of AME treatment increased the protein levels of FGF21, CTSB, and irisin. Both doses of AME treatment increased the protein level of BDNF (Figure 5C).

In the hippocampus of T2DM mice, AME supplementation increased the protein levels of FGFR1 at the high dose and β-klotho regardless of the dose. Both doses of AME treatment increased the protein level of BDNF. In addition, the high dose of AME treatment increased the protein level of CTSB (Figure 5D).

## 4. Discussion

In this study, we demonstrated the beneficial effects of AME treatment on the multi-organ energy metabolism associated with muscle–brain connectivity in T2DM. Specifically, we focused on myokines released by skeletal muscle such as BDNF, irisin, and CTSB which contribute to brain physiological and metabolic responses. A recent study suggests that the myokines can act to regulate neurogenesis and angiogenesis, which contribute to brain metabolism. In our previous study, we confirmed the mechanisms connecting peripherally released myokines and hepatokines to brain function in T2DM [8]. In addition, increasing evidence indicates that abnormal peripheral homeostasis influences brain metabolism and cognitive function [8,9]. Therefore, the regulation of liver/muscle-initiated signaling molecules in T2DM can facilitate innovative therapeutic approaches.

A major characteristic in T2DM is abnormal glucose homeostasis in the brain and peripheral tissues including those of the liver and skeletal muscle [10]. Chronic hyperglycemia-mediated metabolic abnormalities lead to the dysfunction and failure of various organs. In this study, AME had regulatory effects on glucose homeostasis in T2DM mice. According to the HPLC analysis, the AME contained various compounds such as rutin, quercetin, kaempferol, annonacin, and annonacinone, which are known to have anti-diabetic effects [20]. At the molecular level, the tyrosine phosphorylation of IRS-1 by insulin activates pAkt and then enhances the translocation of GLUT to increase glucose uptake [26]. Under diabetic conditions, increased serine/tyrosine phosphorylation of IRS-1 inhibits the insulin signaling pathways, resulting in IR. This study showed that AME treatment selectively enhanced insulin signaling pathways in the liver, skeletal muscle, and hippocampus. However, the effect of AME in hepatic tissue in T2DM mice was not strongly significant compared to the results of our previous study [20]. It might be caused by the greater individual difference and different baseline condition in T2DM compared to that of the previous study, which might have led to the disappearing significance between the groups in the result. In this study, we analyzed the ratio of serine phosphorylated IRS-1/total IRS-1, which is more specifically associated with a negative regulator of insulin signaling. From this result, the insulin signaling pathways in each tissue might partially contribute to regulate FBG in T2DM mice. The evidence showed that kaempferol, one of the components of AME, stimulated glucose absorption in the skeletal muscle of rats [27]. Furthermore, dietary quercetin attenuated IR by increasing PGC1α expression in skeletal muscle [28,29]. These results demonstrate that AME supplementation might have a crucial role in glucose regulation under T2DM conditions due to the synergistic or additive effects of active ingredients in AME. Based on the results, we can suggest that AME might be useful in reducing multi-organ IR, which is a major risk factor for multi-organ damage in T2DM.

Impaired insulin signaling affects energy metabolism [30]. Aberrant energy metabolism in skeletal muscle and hepatic tissue influences the releases of myokines and hepatokines, respectively, which are crucial to maintain whole-body energy homeostasis [11,16]. Particularly, FGF21 is a hormone that regulates energy metabolism via the muscle–liver axis [31,32,33,34]. FGF21 increases cellular nicotinamide adenine dinucleotide+ levels, leading to the activation of SIRT1, which enhances PGC1α activity [35]. PGC1α promotes mitochondrial replication and biogenesis, which regulate energy production [35]. PGC1α also enhances glucose absorption to each cell by increasing the GLUT translocation, thereby reducing IR [36]. A previous study demonstrated that quercetin, abundant in AME, modulated AMPK/SIRT1 signaling in a rodent model of atherosclerosis [37]. Furthermore, rutin activated skeletal muscle AMPK with increased mitochondrial biogenesis in HFD-induced obese rats [38]. In the present study, AME treatment enhanced FGF21-dependent SIRT1/PGC1α pathways in the liver and skeletal muscle, although the markers were selectively modulated with different treatment doses in T2DM mice. As principal organs are responsible for glucose disposal and energy homeostasis, the liver and skeletal muscle have key roles that can impact the core pathogenesis of a systemic metabolic disease via the liver–muscle axis. Consequently, regulation of the energy balance in these tissues is crucial for whole-body energy expenditure. According to the results of this study, AME has beneficial effects on protecting against diabetes-related aberrant peripheral energy metabolism through activation of the PGC1α/SIRT1-dependent mechanism by FGF21.

IR with deficient energy stimulates protein breakdown by activating the ubiquitin-proteasome proteolytic pathway [39]. Reduced Akt activates transcription factor Forkhead box class O 3a (FOXO3a) which translates muscle-specific ubiquitin ligase. It promotes transcription of the muscle RING-finger 1 (Murf1) and atrophy-related ubiquitin ligases atrogin-1/MAFbx [40]. In this study, AME supplementation reduced the protein levels of Murf1 and Atrogin-1 along with nuclear Foxo3a in diabetic skeletal muscle. In accordance with this result, AME treatment recovered the muscle cross-sectional area that was decreased in the DMC group. Therefore, it can be concluded that AME has beneficial effects on diabetic muscle alteration by maintaining the myofiber size and reducing muscle degradation.

On the other hand, enhanced energy metabolism by AME treatment contributes to increased liver-derived FGF21 and muscle-derived FGF21, CTSB, BDNF, and irisin. These hepatokines/myokines are known to enhance hippocampal neurogenesis by passing through the BBB [41,42,43]. In plasma, AME supplementation at the high dose upregulated the levels of endocrine factors including FGF21, CTSB, irisin, and BDNF in T2DM mice. It postulates that increased hepatokines/myokines by AME supplementation might potentially contribute to neuronal function. In addition, activated PGC1α in skeletal muscle leads to the biosynthesis of KYN aminotransferase (KAT), an enzyme that converts neurotoxic KYN into neuroprotective kynurenine acid (KYNA), thereby suppressing toxic accumulation in the brain [44,45]. AME treatment decreased the protein level of skeletal muscle KYN, which reroutes KYN metabolism to increase energy efficiency in T2DM mice.

Based on these results, we examined hippocampal neuroprotection markers via energy metabolism. The protein levels of hippocampal BDNF and CTSB were downregulated in T2DM mice. BDNF is one of the neurotransmitter modulators that protects neurons and improves the function of CNS [46]. Reduced hippocampal BDNF is highly correlated with cognitive impairment in diabetes [47]. Administration of BDNF improved energy expenditure in the hypothalamus of db/db mice [48]. In addition, CTSB has been considered to protect ß-amyloidosis (Aβ) and cognitive function in Alzheimer’s disease models [49]. CTSB promotes Aβ degradation by catalyzing the cleavage of Aβ peptides [50]. In this study, AME supplementation enhanced the protein levels of CTSB at the high dose and BDNF at both doses in T2DM mice. In addition, the hippocampal FGF21 level was not different among the groups, but AME supplementation enhanced the protein levels of FGFR1 and β-klotho, which determines the sensitivity of organ-to-FGF21 signaling in T2DM. It is important because FGF21 metabolism is modulated by the presence of FGFR1 and β-klotho. In this context, AME has a neuroprotective effect which contributes to reduce vulnerability of the brain causing cognitive impairment.

Furthermore, the AMPK/PGC1α axis has a role in the survival and death of neurons to maintain cellular metabolic homeostasis [51,52]. Mitochondrial function with diminished reserve capacity was impaired with inhibited AMPK/PGC-1α pathways in diabetes [53]. In particular, AMPK is involved in autophagy, which degrades cell debris including proteins and organelles to maintain the energy balance [5,47]. AMPK stimulates autophagy by inactivating mTOR and increasing the LC3 expression [54]. In the results of this study, AME treatment enhanced the AMPK/PGC-1α pathways and mTOR-autophagy in T2DM mice. Similarly, quercetin attenuated diabetic neuropathy by regulating the mitochondrial AMPK/PGC1α pathway in vivo and in vitro [55]. Therefore, it can be suggested that AME has a neuroprotective effect by the regulation of cellular energy homeostasis via hippocampal autophagy-related markers in diabetic conditions. In accordance with the above results, AME treatment decreased morphological impairments such as nucleic pyknosis and neuron damage in T2DM mice.

Collectively, this study demonstrated that molecular pathways can be selectively regulated at different doses of AME and that the most effective dose of AME treatment might be tissue-specific in T2DM.

The important point is that the pattern of hippocampal CTSB and BDNF showed similar trends to those in the plasma and skeletal muscle. It can be postulated that improved peripheral energy metabolism via the liver–muscle axis by AME treatment might contribute to neuroprotection mediated by brain function-related myokines in T2DM. In addition, each myokine derived from skeletal muscle makes an additive or synergistic contribution to neuronal function. Although it has not been shown that myokines derived from skeletal muscle effectively cross the BBB and stimulate neurotrophins, for the first time, this study suggests the possibility that myokines induced by AME act peripherally to promote metabolic and physiological changes with consequences in the brain.

As there is a lack of an effective method for targeting inter-organ crosstalk exploration, multi-organ crosstalk mechanisms associated with metabolic regulation were briefly investigated. Further study using proteomics or in vivo tracer techniques might provide more convincing evidence for the multi-organ crosstalk signals. Additionally, further network analysis would be helpful to enhance research on organ crosstalk as a potential therapeutic strategy in T2DM.

## 5. Conclusions

The present study demonstrated that AME treatment improved the insulin-signaling and FGF21-dependent energy metabolism pathway in the liver, skeletal muscle, and hippocampus of T2DM mice. Particularly, AME supplementation increased brain function-related myokine/hepatokine levels in the skeletal muscle and liver, respectively, showing similar patterns to those of plasma. In addition, AME treatment increased neuroprotective markers in T2DM mice. In conclusion, AME could be a potential nutraceutical for improving aberrant organ energy metabolism with muscle–brain connectivity via brain function-related myokines in T2DM. Further study is needed to identify the multi-organ crosstalk and clarify the ameliorative effects of dietary intervention on multi-organ targeting mechanisms as potential therapeutic strategies in T2DM.

## Figures and Tables

**Table 1:** Effects of AME treatment on body composition, food intake, and glycemic regulation in T2DM mice.

GROUP	CON	DMC	LAM	HAM
Body weight (g)				
Before treatment	26.76 ± 0.50 ^a^	34.87 ± 0.77 ^b^	33.91 ± 1.22 ^b^	34.69 ± 0.72 ^b^
After treatment	30.40 ± 0.67 ^a^	40.20 ± 1.27 ^b^	39.82 ± 1.37 ^b^	40.94 ± 1.46 ^b^
Gain	3.20 ± 0.39 ^a^	4.66 ± 0.48 ^ab^	4.67 ± 0.62 ^ab^	5.56 ± 0.63 ^b^
% Fat	31.61 ± 2.04 ^a^	42.40 ± 3.7 ^b^	46.10 ± 2.04 ^b^	45.43 ± 2.77 ^b^
% Lean	65.31 ± 2.21 ^b^	55.16 ± 3.90 ^a^	51.08 ± 2.04 ^a^	52.24 ± 2.94 ^a^
Gastrocnemius weight (g/kg BW)	0.54 ± 0.01 ^b^	0.43 ± 0.01 ^a^	0.43 ± 0.02 ^a^	0.42 ± 0.02 ^a^
Quadriceps weight (g/kg BW)	0.63 ± 0.07 ^b^	0.43 ± 0.01 ^a^	0.43 ± 0.05 ^a^	0.48 ± 0.02 ^a^
Food intake (g/day)	3.35 ± 0.01	3.25 ± 0.08	3.41 ± 0.03	3.28 ± 0.07
Fasting blood glucose level (mg/dl)				
4 weeks after treatment	163.64 ± 7.37 ^a^	331.18 ± 18.28 ^c^	269.78 ± 9.91 ^b^	336.56 ± 23.67 ^c^
9 weeks after treatment	146.36 ± 4.55 ^a^	291.64 ± 26.97 ^c^	235.33 ± 7.00 ^b^	282.33± 20.67 ^bc^
Plasma insulin (μU/mL)	10.00 ± 1.23 ^a^	22.49 ± 5.36 ^b^	9.52 ± 2.68 ^a^	14.14 ± 3 ^ab^
HbA1c%	5.01 ± 0.07 ^a^	5.94 ± 0.23 ^b^	5.25 ± 0.17 ^a^	5.18 ± 0.21 ^a^
HOMA-IR (mmol/L × μU/mL)	3.65 ± 0.51 ^a^	16.01 ± 3.6 ^b^	5.74 ± 1.79 ^a^	10.08 ± 2.41 ^ab^

Values are means ± SEM. There is no significant difference between values with the same superscript letters in the row (n = 9 per group).

## Data Availability

The data presented in this study are available on request from the corresponding author. The data are not publicly available due to privacy. The studies not involving humans.
